# Conditioned Pain Modulation in Patients with Hemophilic Arthropathy: A Cross-Sectional Cohort Study

**DOI:** 10.3390/jcm14051728

**Published:** 2025-03-04

**Authors:** Mario Bermúdez-Egidos, Raúl Pérez-Llanes, Roberto Ucero-Lozano, Rubén Cuesta-Barriuso

**Affiliations:** 1 Department of Physiotherapy, University of Murcia, 30120 Murcia, Spain; mario.bermudeze@um.es (M.B.-E.); rperez@um.es (R.P.-L.); 2 InHeFis Research Group, Instituto Asturiano de Investigación Sanitaria (ISPA), 33011 Oviedo, Spain; cuestaruben@uniovi.es; 3 Department of Physiotherapy, Faculty of Medicine, Health and Sports, European University of Madrid, 28670 Madrid, Spain; 4 Department of Surgery and Medical-Surgical Specialties, University of Oviedo, 33006 Oviedo, Spain

**Keywords:** hemophilia, pain, chronic pain, conditioned pain modulation, anxiety, kinesiophobia

## Abstract

**Background/Objectives**: Hemophilic arthropathy causes functional impairment, disability, and chronic pain. Conditioned pain modulation describes the effect of endogenous pathways that potentiate or diminish the effects of noxious afferent stimuli. The objective was to identify conditioned pain modulation in patients with bilateral hemophilic ankle and knee arthropathy, and the best predictive model thereof. **Methods**: Cross-sectional cohort study. Forty-nine adult patients with hemophilic arthropathy were recruited. The dependent variable was the Conditioned Pain Modulation Index (CPMI). Age was the predictor variable. Secondary variables, estimated as modifying or confounding variables, were kinesiophobia (Tampa Scale for Kinesiophobia), catastrophizing (Pain Catastrophizing Scale), anxiety (State-Trait Anxiety Inventory), and clinical, anthropometric, and sociodemographic variables. **Results**: Conditioned pain modulation in patients with hemophilic arthropathy presents values close to zero (mean = 0.004: 95%CI: −0.05; 0.06). Anxiety, pain intensity, and pressure pain threshold explained the variability in the conditioned modulation of ankle pain (R^2^_adj_ = 0.24). Variables explaining 23.05% of variability of conditioned modulation of knee pain were age, inhibitor development, anxiety, and pressure pain threshold (R^2^_adj_ = 0.23). **Conclusions**: Patients with hemophilia presented a modulation close to zero, representing a balance between the ability to inhibit and facilitate painful stimuli. The predictive model of conditioned modulation of ankle pain includes anxiety, and pain intensity and threshold. Age, inhibitory development, anxiety, and pain threshold predict knee pain modulation.

## 1. Introduction

Chronic joint pain is one of the main characteristics of hemophilic arthropathy [1]. Pain, both acute pain characteristic of hemarthrosis and chronic pain secondary to hemophilic arthropathy, tends to generate sensitization to noxious stimuli and hyperexcitability to non-painful stimuli [2]. Chronic pain is clinically more complex, as it remains active over time. The establishment of this pain causes excessive activity of the nociceptive system that maintains chronic pain and complicates the effectiveness of treatment [3]. These alterations can diminish the efficacy of analgesic treatments in patients with chronic pain [4].

Pain management in patients with hemophilia varies depending on the presence of bleeding. In the case of hemarthrosis, the only effective treatment for pain relief is the early and continuous administration of the missing clotting factor to stop the bleeding. However, when the pain is associated with joint damage, a multidisciplinary approach is more appropriate. Radiosynovectomy and chemical synovectomy can reduce pain in these patients by inhibiting the vicious circle of hemarthrosis–synovitis–hemarthrosis [5]. When pain is secondary to severe hemophilic arthropathy, the physiotherapeutic approach using manual therapy techniques and therapeutic exercise has proven effective in this population [6]. However, when joint deterioration is very advanced and pain cannot be mitigated, the most commonly used resources are arthrodesis and total arthroplasty, which have proven effective in reducing knee and ankle pain [7,8].

For the management of chronic pain due to advanced hemophilic arthropathy through conservative treatment with physiotherapeutic techniques, isometric, isotonic and proprioceptive exercises, stretching, educational sessions, and home exercise have been described as effective. In the surgical treatment of chronic pain, joint replacement is the last option to recover function. Surgical treatment is usually more common on ankles and knees.

Pain modulation is the ability of the central nervous system to influence the pain threshold by processing somatosensory inputs in the spinal cortex, which may decrease or facilitate pain [9]. It has been observed that in patients with knee osteoarthritis and chronic pain, there is a decrease in the efficacy of this modulation [10].

Deficits in pain inhibition mechanisms are assessed by conditioned pain modulation [11]. This assessment is based on the fact that pain intentionally caused in a specific body region (conditioning stimulus) decreases the pain perceived in a different region (conditioned stimulus) [12]. The nociceptive stimuli used to test pain modulation are varied: cold, heat, ischemia, and tolerated pain thresholds; fixed-intensity supra-threshold stimulus; and spatial summation in different body regions [13]. Similarly, the relationship between conditioned pain modulation and central sensitization has been observed in patients with knee osteoarthritis and chronic pain [14]. Chronic pain triggers an alteration and dysfunction in endogenous pain inhibition mechanisms [15]. This deterioration of the descending pathways in patients with chronic pain may play a part in triggering central sensitization. As well as regulating mood, the prefrontal lobe is responsible for the processing of nociceptive stimuli and descending pathways. Therefore, it is necessary to assess anxiety in central sensitization and in conditioned pain modulation [16].

Pain management in hemophilia patients is often inadequate, with a risk of developing chronic pain that is difficult to manage. The fundamental pillars of the approach to pain in these patients have been described on numerous occasions: maintaining suitable coagulation, systemic pain therapy, exercise and physiotherapy, and psychological care [2]. However, it has been reported that up to 67.6% of patients were very dissatisfied or dissatisfied with their overall pain management [17]. Accordingly, the aim of this study is to describe conditioned pain modulation in patients with bilateral hemophilic knee and ankle arthropathy, and to identify the best predictive model of conditioned pain modulation in these patients.

## 2. Materials and Methods

### 2.1. Study Design

Cross-sectional cohort study.

### 2.2. Participants

Patients were recruited between May and June 2024 from five regions of Spain through the Spanish Federation of Hemophilia.

Inclusion criteria were patients with a diagnosis of hemophilia A and B; with severe hemophilia phenotype; over 18 years of age; with a medical diagnosis of bilateral hemophilic ankle arthropathy; with clinical assessment by the Hemophilia Joint Health Score (>4 points); on demand or prophylactic treatment with FVIII concentrates/clotting FIX or monoclonal bispecific antibodies; and having signed the informed consent document.

The exclusion criteria were patients with neurological or cognitive disorders that prevented them from understanding the questionnaires and physical tests; patients who had presented ankle hemarthrosis in the 6 months prior to the start of the study; patients who had taken analgesic or anti-inflammatory drugs in the 30 days prior to the study; and those patients undergoing an intervention (physiotherapeutic or orthopedic) at the time of the study.

The non-inclusion of patients undergoing analgesic drug treatments, physiotherapy, or surgery was due to the possible influence of these factors. Greater changes in pain are obtained in patients with a good conditioned pain modulation taking non-steroidal anti-inflammatory drugs [18].

The combination of physiotherapy techniques such as thermotherapy and electrotherapy does not appear to cause significant changes in endogenous pain inhibitory function, but it does affect pressure pain thresholds [19]. Similarly, when conditioned pain modulation is inefficient in people with knee osteoarthritis before undergoing joint replacement, they experience more postoperative pain. Thus, patients with a deficit of preoperative endogenous analgesia suffer from persistent postoperative pain 6 months after the intervention [20].

### 2.3. Ethics Considerations

All hemophilia patients included in the study were informed of the objectives of the study. All patients gave their approval, signing the informed consent document. This study complied with the regulations, in accordance with the Helsinki guidelines. The Research Ethics Committee of the University of Murcia (ID: M10/2024/128, 21 March 2024) approved the study. The study was registered in the Protocol Registration and Results System (www.clinicaltrials.gov; ID: NCT06267209; accessed on 2 January 2025).

### 2.4. Measurement Instruments

The evaluation was carried out at the premises of the hemophilia associations included in the study. All the evaluations were performed by the same physical therapist, following the same evaluation protocol.

**Figure 1:**
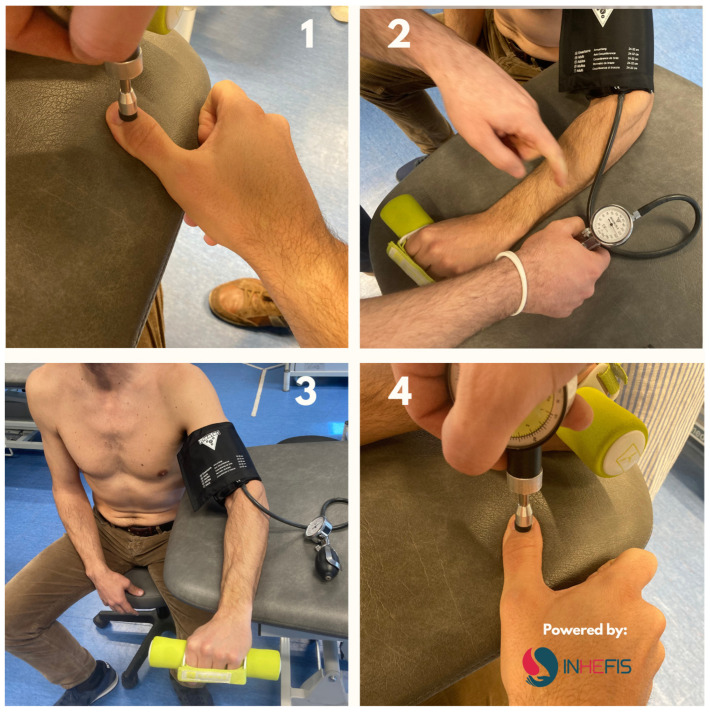
Evaluation process of the CPM with pressure algometry. 1: PPT measured at the base of the dorsal part of the distal phalanx of the thumb; 2: Ischemia test on the contralateral upper limb. Sphygmomanometer inflated to 240 mmHg; 3: Resistance exercises in wrist extension, (2 kg lifting). Maximum 45 repetitions or pain level > 7/10 (NRS); 4: Sphygmomanometer still inflated, PPT repeated.

The primary variable of this study was conditioned pain modulation, with age as the dependent variable. The secondary variables, estimated as modifiers or confounders, were kinesiophobia, catastrophizing, perceived anxiety, joint damage, pain intensity and pain threshold to pressure, and type of treatment and development of inhibitors. The measurement instruments employed to measure these variables were as follows:

- The Conditioned Pain Modulation Index was used to evaluate the endogenous pain inhibition by means of the facilitation or inhibition of responses to a conditioned stimulus. To perform an assessment of the diffuse descending inhibitory system, tonic pain was elicited by exerting pressure on a non-painful area [21]. First, the pressure pain threshold (PPT) was measured at the base of the dorsal part of the distal phalanx of the thumb, and then a conditioned stimulus was elicited using the ischemia test on the contralateral upper limb. For the ischemia test, a sphygmomanometer was placed on the arm about 14 cm from the cubital fossa, inflated to 240 mmHg. Patients were asked to indicate pain intensity on a numerical pain rating scale of 0–10 until a pain of 7/10 appeared. Subsequently, patients performed resistance exercises in wrist extension, lifting a 2 kg weight up to a maximum of 45 repetitions or until a pain level greater than 7/10 was reached. At that time, with the sphygmomanometer still inflated, and pain threshold measurements were repeated at the dorsal part of the distal phalanx of the thumb. The sphygmomanometer was kept inflated between zero and six minutes to reach the pain intensity score of 7/10. The time was not extended beyond six minutes, as with this type of stimulation there is more activation to a greater magnitude of pain but not to a longer duration of this type of conditioned stimulus [21]. Figure 1 shows the process of evaluating conditioned pain modulation using a pressure algometer.

Once these data were obtained, the transformation to a Conditioned Pain Modulation Index score was calculated using the following formula:

$$\mathrm{CPMI}=\frac{{\mathrm{PPT}}_{\mathrm{post}}-{\mathrm{PPT}}_{\mathrm{pre}}}{{\mathrm{PPT}}_{\mathrm{pre}}}$$

CPMI: Conditioned Pain Modulation Index.

PPT: Pressure Pain Threshold.

- The Spanish version of the Tampa Scale of Kinesiophobia (TSK-11SV) [22] was used to assess fear of movement in the patients included in the study. This tool consists of 11 items. This scale has shown high reliability (ICC = 0.87) [23]. Its values range from 11 to 44 points, where the higher the score, the greater the fear of movement.
- The Spanish validated version of the Pain Catastrophizing Scale [24] was used to evaluate catastrophizing in patients with hemophilia. This scale is composed of 13 items and has shown high reliability (ICC = 0.84) [25]. It is rated within a range of 0 to 52 points, where a lower score indicates less catastrophizing.
- Patients’ perception of anxiety was measured with the Spanish version of the State-Trait Anxiety Inventory (STAI) [26]. This tool consists of 20 items. This scale has shown high reliability (ICC = 0.84) [27]. This scale evaluates the state and trait anxiety of each subject with a score of 0 to 30 points for each scale, where a higher score indicates a higher rate of trait or state anxiety.
- Joint damage was assessed using the Hemophilia Joint Health Score [28]. This tool is suitable for the identification of joint alterations in children, adolescents, and adults with hemophilia [29]. This scale has shown good reliability (ICC > 0.70) [30]. It scores from 0 (no joint damage) to 20 points (maximum joint damage) per joint. In the evaluation of total joint damage, a gait assessment (range 0–4 points) is added to the 120 points, with the maximum assessment of this scale being 124 points.
- The Visual Analog Scale [31] was used to measure the pain intensity perceived by the patients. This scale assesses the patients’ perception of pain ranging from 0 to 10 points. A score of 0 indicates that the patient does not perceive pain, with the maximum score corresponding to the maximum pain suffered or imaginable.
- Functional capacity was measured with the 6-Minute Walk Test (6-MWT) [32]. This test is performance-based, measuring walking speed and submaximal exercise capacity. Patients are instructed to walk a 30 m track for 6 min and try to cover as much distance as possible without running. This scale has shown excellent reliability (ICC = 0.97) [33]. The distance covered, in meters, was recorded, allowing the use of a walking aid or orthosis.
- Pressure pain threshold was assessed with a pressure algometer (Wagner FDIX model, Wagner Instruments, Riverside, CT, USA) [34]. Pressure was applied bilaterally and caudal to the lateral malleolus and medial malleolus [35]. The pressure applied by the evaluator was increased at a rate of approximately 50 kPa/s until the patient indicated that the sensation was becoming painful [36]. This tool has shown excellent reliability (ICC= 0.98–0.99) [34]. The unit of measurement is the Newton.

The main sociodemographic (age, marital status, educational level, and employment status), clinical (type of hemophilia, pharmacological treatment and development of inhibitors), and anthropometric (weight, height and body mass index) variables were collected.

Prior to patient recruitment, a pilot study was carried out on the intra-observer reliability of the Conditioned Pain Modulation Index. Seven patients with hemophilia, not included in the study, were evaluated on two consecutive days by the study evaluator, under the same conditions and following the same protocol. High intra-observer reliability was found in the evaluation of conditioned pain modulation (ICC = 0.91).

### 2.5. Sample Size

The sample size was calculated with the G*Power statistical package (version 3.1.9.2; Heinrich-Heine-Universität Düsseldorf, Düsseldorf, Germany) based on the development of a multiple regression analysis model, with a level α = 0.05 and a statistical power of 80% (1-β = 0.80). Thus, for the five Spanish regions where the study patients were recruited, a sample of 51 patients with hemophilia was estimated. The sample size of this study was not adjusted for possible dropouts or missing data.

### 2.6. Statistical Analysis

Statistical analysis was performed with version 17.0 of the STATA statistical package for Windows (Stata Corp LP, College Station, TX, USA). The intra-rater reliability analysis was carried out using the two-factor random intraclass correlation coefficient. The main descriptive statistics of central tendency and dispersion (mean and standard deviation) of the variables evaluated were obtained.

This method of analysis constructs all the possible sub-models by combining the terms of the maximum model, evaluating the degree of compliance with the established criteria for each of the models: smaller Mallows’ Cp and higher adjusted R^2^. The variance inflation factor (VIF) was used to calculate the multicollinearity of the predictor variables. A cross-validation was carried out, calculating the goodness-of-fit measure, to obtain the predictive capacity of the model. To calculate the outlying values, the standardized distance between the subject and the point defined by the mean of all the predictor variables (Mahalanobis distance) was calculated, with Cook’s distance being the main indicator of influence used.

Normality was verified by applying the Shapiro–Wilk test to the raw residuals, while homogeneity of variances was verified with the Breusch–Pagan test. To estimate the effect of the model, the modifying and confounding effect of the independent variables was assessed, with age as the adjustment variable.

Using Student’s *t*-test for independent samples, we assessed whether there were differences in conditioned pain modulation based on the type of treatment, the type of hemophilia, or the development of inhibitors. In this study, the significance level was α < 0.05.

## 3. Results

The mean age of the 49 patients with hemophilia included in the study was 43.29 (SD: 10.24) years, with a mean body mass index of 27.21 (SD: 3.60) kg/m^2^. Most patients had hemophilia A (85.71%), and only 24.49% of patients had developed antibodies to FVIII/FIX clotting concentrates. Patients exhibited conditioned pain modulation values of 0.004 (SD: 0.28; 95%CI = −0.05; 0.06). Table 1 shows the main descriptive characteristics of the patients included in the study.

### 3.1. Analysis of the Diagnosis of the Selected Model in Ankle Arthropathy

The predictive model of conditioned modulation of ankle pain was analyzed. The best model included the variables of state anxiety, trait anxiety, ankle pain intensity and pressure pain threshold (C_p_ = 2.30), explaining 24.05% of the variability in conditioned pain modulation (R^2^_adj_ = 0.51). When performing multicollinearity analysis of the independent predictor variables, we observed how the variables are moderately correlated with each other (mean VIF = 2.48). The VIF values corresponding to the variables show that there is no collinearity issue. The external validity analysis evidenced that the model is quite reliable, with a loss of predictive capacity of 8.84% (R^2^-r^2^ = 0.088). The internal validity of the model (R^2^-mean = 0.21) was calculated as the predictive ability of the model when run on external samples. When calculating the loss of predictive ability, a highly generalizable model was obtained (3.05% < 10%; Adj R-squared = 0.24) where only seven subjects (14.28%) showed extreme residual values and distant values (Lever > 0.10; Mahalanobis distance > 9.42). The normality assumption was met (*p* = 0.11), with heterogeneity in the dispersion of the residuals (χ^2^_(1)_ = 8.42; *p* = 0.003). Table 2 shows the multiple linear regression model for the conditioned modulation of ankle pain.

### 3.2. Analysis of the Diagnosis of the Selected Model in Knee Arthropathy

In the analysis of the best predictive model of the conditioned modulation of knee pain, the best model included the variables age, inhibitor development, state anxiety and pressure pain threshold (C_p_ = 2.24), explaining 23.05% of the variability in the conditioned modulation of knee pain (R^2^_adj_ = 0.23). When multicollinearity analysis was performed, the variables were moderately correlated with each other (mean VIF = 1.16). The small VIF values corresponding to the variables show that there is no collinearity problem. In the analysis of external validity, the model was shown to be very reliable with a loss of predictive ability of 0.6% (R^2^-r^2^ = 0.0059). The internal validity of the model (R^2^-mean = 0.17) was calculated as the predictive ability of the model when run on external samples. When calculating the loss of predictive ability, a generalizable model was obtained (6.12% < 10%; Adj R-squared = 0.23) where only four subjects (8.16%) showed extreme residual values, and six patients presented distant values (Lever > 0.10; Mahalanobis distance > 9.2). There was no significant violation of normality in the raw residuals (*p* = 0.06), finding heterogeneity in the dispersion of the residuals (χ^2^_(1)_ = 8.79; *p* = 0.003). Table 3 shows the multiple linear regression model for the conditioned modulation of knee pain.

### 3.3. Analysis of Differences in Means for Independent Samples

When comparing conditioned pain modulation according to clinical variables, we observed statistically significant differences based on the type of treatment (t = −3.11; *p* = 0.002). However, there were no statistically significant differences in conditioned pain modulation according to the type of hemophilia (t = 1.25; *p* = 0.21) or the development of inhibitors (t = 1.85; *p* = 0.07). Table 4 shows the results of the independent samples’ mean comparison analysis.

## 4. Discussion

The present study aimed to describe conditioned pain modulation in patients with bilateral hemophilic knee and ankle arthropathy and to identify the best predictive model of this modulation. In the patients included in our study, we observed a modulation close to zero, which shows a balance between the capacity to inhibit and to facilitate painful stimuli. At lower state anxiety and higher trait anxiety, pain intensity, and pressure pain threshold, there was an improved pain modulation and inhibition in hemophilic ankle arthropathy. However, in knee arthropathy, pain inhibition was greater in older patients, who had not developed inhibitors, with a lower state anxiety and a higher pain threshold.

Positive values of conditioned pain modulation represent the patient’s ability to inhibit painful stimuli, while negative values facilitate these stimuli. Conditioned modulation is a system whereby pain inhibits pain. It acts when the intensity of a painful stimulus is diminished by the processing of another painful stimulus. Conditioned pain modulation is a powerful analgesic process. This process occurs due to the presence of active circuits in the brain stem during the management of acute noxious stimuli related to analgesic responses mediated by conditioned pain modulation [37]. Pain modulation occurs at primary afferent synapse levels, in regions such as the caudal division of the spinal trigeminal nucleus, dorsal reticular subnucleus, and parabrachial nucleus. This neural activity can also develop at higher levels, such as the prefrontal and cingulate cortex, when nociceptive stimuli are elicited. All these regions are involved in analgesia resulting from conditioned pain modulation [38].

In this study, we note that the values of conditioned pain modulation in hemophilia patients are close to zero. These results, where there is no clear tendency to facilitate or inhibit pain, may be due to the central sensitization suffered by patients with hemophilic arthropathy [39]. In patients with knee osteoarthrosis, pain hypersensitivity, medullary sensitization, and amplification of signals secondary to active descending pain-facilitating pathways are related to alterations or dysfunction in conditioned pain modulation [40]. In patients with knee osteoarthritis and chronic pain, dysfunctional conditioned pain modulation is related to the increase in delta activity in frontal and central brain regions, areas that control pain and the descending inhibitory system of pain [41]. Conditioned pain modulation is also altered in patients with fibromyalgia. These patients present more powerful responses to nociceptive stimuli and show lower processing of descending pain mechanisms. However, it has been described that sex, age, and pain catastrophizing can vary in the CPM response [12].

Thus, repeated stimuli provoked in the descending pain inhibitory pathways in pathologies characterized by chronic pain may alter their functioning [42]. Thus, central sensitization in patients with chronic pain may reduce the performance and expected results of conditioned pain modulation [14].

Anxiety behaves as a prospective anticipatory emotion that predisposes the subject to face upcoming situations or events. A distinction can be made between anticipatory emotions and anticipated emotions. Anticipatory emotions are those that have already been experienced in relation to a future event (emotional anxiety when thinking about an upcoming event). Anticipated emotions are those that are expected when the subject faces the future event (emotional state during the upcoming event) [43]. Our results show how trait anxiety behaves as a protective factor, where the higher the trait, the better the modulation of pain. On the contrary, the patient’s state anxiety behaves as a risk factor decreasing conditioned pain modulation in patients with hemophilia

Trait anxiety is a state that predisposes a person to perceive non-dangerous situations in a hostile manner. This results in a tendency to avoid anxiety-generating events and elevated basal physiological activation states with a sense of vulnerability. Patients with high trait anxiety develop different responses and information processing than individuals with low levels. Thus, they are conditioned by the attentional focus on threat. This trait anxiety is characterized as a state of hyperarousal and constant hypervigilance that incites the desire to avoid situations perceived as dangerous [44].

State anxiety causes subjective feelings of apprehension, it is an anxious state and a hyperexcitation of the autonomic nervous system. People with elevated levels of anxiety generally mismanage fear and can either maintain it for a long period of time or make it non-existent. High levels of anxiety sensitization in individuals with elevated trait anxiety explain how they may perceive harmless physical symptoms as threatening [45]. This creates an added vulnerable state with which they may develop anxiety-related disorders such as panic. This explains how increased anxiety can negatively condition the processing of different stimuli. Accordingly, good conditioned pain modulation needs state anxiety levels to be as low as possible [46]. In addition, patients who have anxiety and/or depression associated with chronic pain may favor the proliferation of proinflammatory cytokines, amplifying pain and hypersensitivity [47].

A decreased pressure pain threshold in ankles, knees, and elbows has been described [48] in patients with hemophilic arthropathy involving joint pain and damage. This is because more deteriorated joints with arthropathy have greater sensitization to pain. By evaluating the pressure pain threshold and pain intensity, we have observed how these variables behave as a protective factor, where the higher the pressure and pain intensity threshold, the better the conditioned pain modulation. People with hemophilia and arthropathy can present acute and chronic pain, secondary to hemarthrosis and hemophilic arthropathy, respectively. This translates into a complex evaluation and control of pain. In patients with chronic pain, endogenous mechanisms of descending pain inhibition that would contribute to the chronic presence of pain may be altered [49]. Decreased pressure pain threshold indices are indicative of a facilitation of central pain modulation mechanisms. Conversely, normal values of pressure pain threshold indicate the proper functioning of conditioned pain modulation. In the presence of an elevated pressure pain threshold, conditioned pain modulation is more efficient [50], as noted in our study. Conditioned pain modulation reflects the capacity for endogenous pain modulation. If the tissue remains constantly sensitized, it appears that this endogenous modulation is not sufficiently inhibiting (or not efficiently inhibiting) sustained stimuli in patients with low PPTs. For this reason, they present higher levels of joint damage due to the pain itself and, a priori, we might say that this correlation is plausible.

When the sensitivity to a pressure stimulus of a patient with hemophilic arthropathy changes towards more normative values, we must understand that something is happening beyond joint degeneration, which is maintained. These changes will always involve the central nervous system as an interpreter of information and the endogenous pain modulation system. This does not mean that changes are not caused in the periphery that lead to this. Neurogenic inflammation implies that the nervous system is capable of generating by itself in order to try to protect a tissue [51]. This inflammation occurs in the periphery but is mediated at a central level. Therefore, when we talk about improved CPM, as an indicator of the functioning of the endogenous pain inhibition system, we cannot exclude peripheral changes. However, this change is mediated by the central nervous system through the descending inhibitory system.

Similarly, it has been observed that the intensity of pain caused by the conditioning stimulus has an impact on conditioned pain modulation. For example, using a painful thermal stimulus such as immersion of the hand in intense cold can lead to an increase and enhanced effect of conditioned pain modulation in healthy subjects. By increasing the intensity of the applied painful stimulus, an increase in the effect of the descending inhibitory mechanisms of pain was observed. Thus, and consistent with our results, the higher the pain intensity, the better the conditioned pain modulation [52].

It has been observed in patients with knee osteoarthritis that variation in conditioned pain modulation can be a predictor of responses to analgesic treatments [18]. A key aspect to take into account is the effect of patients’ expectations regarding the effects of conditioned pain modulation. In fact, in patients with chronic lower back pain, opioid mechanisms secondary to drug intake do not appear to be involved in the effects of expectation. Hyperalgesic responses are mediated by opioids while hypoalgesic responses are not [53].

### 4.1. Limitations of the Study

The results of this study should be considered with some amount of caution. Firstly, the sample size should be considered as a limitation. Despite being a rare disease, sample selection bias may limit the generalizability of the results to the general population. The fact that hemophilic arthropathy of the elbow has not been evaluated also limits the results, preventing verification of the predictive models of conditioned pain modulation in the upper limb. Evaluation with generic instruments, not specific for patients with hemophilia, can induce evaluation bias, preventing the comparison of results with other studies. This study does not include an evaluation of conditioned pain modulation in patients with moderate and mild hemophilia phenotypes. Likewise, the non-inclusion of patients who regularly take analgesic drugs, as well as the non-measurement of depression as a study variable, makes us cautious about the identified model and its effects.

### 4.2. Recommendations for Clinical Practice

The average values of conditioned pain modulation, close to zero, do not show the presence of inhibition or the facilitation mechanisms in pain modulation in patients with hemophilia. The development of this predictive model of conditioned pain modulation can facilitate a truly comprehensive approach to patients with hemophilia. These results make it possible to identify the influence of pain threshold and intensity, and anxiety on conditioned pain modulation in these patients. In this way, the preventive management of these factors can be promoted in this population. It was noted that the CPM test can be a biomarker for identifying people at high risk of developing chronic pain [54,55]. Therefore, the applicability of the proposed model in routine clinical practice for patients with hemophilia should be considered. Similarly, addressing and controlling anxiety should be a primary objective for pain management in patients with hemophilic arthropathy.

## 5. Conclusions

Adult patients with bilateral knee and ankle arthropathy do not present clear inhibition or facilitation mechanisms in conditioned pain modulation. Pressure pain threshold and intensity and perceived anxiety are the variables that best predict conditioned ankle pain modulation in patients with hemophilic arthropathy. Age, previous development of inhibitors, anxiety levels, and pressure pain threshold predict the conditional modulation of knee pain in patients with hemophilia. The evaluation of conditioned pain modulation in clinical practice can promote the monitoring and control of pain in patients with hemophilia. Future research should confirm these results, including the evaluation of drug intake, physical analgesic interventions, and depression.

## Figures and Tables

**Table 1:** Descriptive analysis of the patients included in the study.

Variable	Mean (SD)	95%CI
Age (years)	43.12 (10.18)	41.07; 45.16
Weight (kg)	81.15 (11.35)	78.87; 83.42
Height (cm)	172.67 (5.96)	171.47; 173.86
Body Mass Index (kg/m^2^)	27.21 (3.58)	26.49; 27.93
Conditioned Pain Modulation Index (0–1)	0.004 (0.28)	−0.05; 0.06
Kinesiophobia (11–44)	25.81 (6.85)	24.44; 27.19
Catastrophism (0–52)	12.85 (12.45)	10.36; 15.35
Anxiety state (0–30)	18.48 (9.20)	16.64; 20.33
Anxiety trait (0–30)	16.83 (12.13)	14.40; 19.26
Functionality (m)	143.90 (32.38)	137.37; 150.43
Ankle joint damage (0–20)	11.63 (2.29)	11.17; 12.09
Ankle lateral malleolus pressure pain threshold (N)	49.25 (18.44)	45.55; 52.95
Ankle medial malleolus pressure pain threshold (N)	52.86 (19.78)	48.89; 56.82
Ankle joint intensity (0–10)	3.09 (2.07)	2.67; 3.51
Ankle range of motion (degrees)	35.38 (14.91)	32.39; 38.37
Knee joint damage (0–20)	10.21 (3.63)	9.48; 10.94
Knee pressure pain threshold (N)	70.87 (31.45)	64.56; 77.17
Knee joint intensity (0–10)	1.21 (1.64)	0.88; 1.54
Knee range of motion (degrees)	110.79 (25.63)	105.65; 115.93
	n	%
Type of hemophilia (A/B)	42/7	85.71/14.29
Treatment (Prophylaxis/On demand)	34/15	69.39/30.61
Development of inhibitors (Yes/No)	12/37	24.49/75.51

SD: standard deviation; 95%CI: 95% confidence interval.

**Table 2:** Multiple linear regression model for conditional modulation of ankle pain.

Conditioned Pain Modulation	Coeff.	*p*-Value	95%CI	VIF
Anxiety state	−0.021	0.000	−0.03; −0.01	3.76
Anxiety trait	0.001	0.032	0.001; 0.02	3.75
Ankle joint intensity	0.03	0.027	0.001; 0.06	1.14
Ankle medial malleolus pressure pain threshold	0.004	0.036	0.001; 0.01	1.26
_constant	0.012	0.914	−0.21; 0.23	

Coeff.: regression coefficient; 95%CI: 95% confidence interval; VIF: variance inflation factor.

**Table 3:** Multiple linear regression model for conditional modulation of knee pain.

Conditioned Pain Modulation	Coef.	*p*-Value	95%CI	VIF
Age	0.004	0.12	−0.001; 0.01	1.13
Development of inhibitors				
Yes	−0.12	0.04	−0.25; −0.002	1.11
Anxiety state	−0.01	0.01	−0.01; −0.002	1.20
Knee pressure pain threshold	0.002	0.02	0.000; 0.001	1.18
_constant	−0.14	0.39	−0.46; 0.18	

Coef.: regression coefficient; 95%CI: 95% confidence interval; VIF: variance inflation factor.

**Table 4:** Comparative analysis of means for independent samples.

Outcomes	Type	Mean (SD)	t	Sig.	95%CI
Type of treatment	On demand	−0.12 (0.28)	−3.11	0.002	−0.30; −0.06
	Prophylaxis	0.06 (0.26)			
Type of hemophilia	Hemophilia A	0.01 (0.27)	1.25	0.21	−0.06; 0.26
	Hemophilia B	−0.08 (0.32)			
Development of inhibitors	Yes	−0.10 (0.37)	1.85	0.07	−0.02; 0.31
	No	0.04 (0.23)			

SD: Standard deviation; Sig.: significance; 95%CI: 95% confidence interval; t: statistic of t-student.

## Data Availability

The data that support the findings of this study are available on request from the corresponding authors. The data are not publicly available due to privacy and ethical restrictions.
